# BERT-m7G: A Transformer Architecture Based on BERT and Stacking Ensemble to Identify RNA N7-Methylguanosine Sites from Sequence Information

**DOI:** 10.1155/2021/7764764

**Published:** 2021-08-25

**Authors:** Lu Zhang, Xinyi Qin, Min Liu, Guangzhong Liu, Yuxiao Ren

**Affiliations:** ^1^College of Information Engineering, Shanghai Maritime University, 1550 Haigang Ave., Shanghai 201306, China; ^2^School of Computer Science and Engineering, Southeast University, Nanjing 214135, China

## Abstract

As one of the most prevalent posttranscriptional modifications of RNA, N7-methylguanosine (m7G) plays an essential role in the regulation of gene expression. Accurate identification of m7G sites in the transcriptome is invaluable for better revealing their potential functional mechanisms. Although high-throughput experimental methods can locate m7G sites precisely, they are overpriced and time-consuming. Hence, it is imperative to design an efficient computational method that can accurately identify the m7G sites. In this study, we propose a novel method via incorporating BERT-based multilingual model in bioinformatics to represent the information of RNA sequences. Firstly, we treat RNA sequences as natural sentences and then employ bidirectional encoder representations from transformers (BERT) model to transform them into fixed-length numerical matrices. Secondly, a feature selection scheme based on the elastic net method is constructed to eliminate redundant features and retain important features. Finally, the selected feature subset is input into a stacking ensemble classifier to predict m7G sites, and the hyperparameters of the classifier are tuned with tree-structured Parzen estimator (TPE) approach. By 10-fold cross-validation, the performance of BERT-m7G is measured with an ACC of 95.48% and an MCC of 0.9100. The experimental results indicate that the proposed method significantly outperforms state-of-the-art prediction methods in the identification of m7G modifications.

## 1. Introduction

RNA posttranscriptional modification (PTM) is a common phenomenon in biological processes [1]. Currently, approximately 170 distinct RNA modifications have been discovered, of which N7-methylguanosine (m7G) is one of the RNA modifications ubiquitous in various species. N7-methylguanosine is a positively charged RNA modification, which is produced by the addition of a methyl group at position N7 of riboguanosine [2, 3]. And its expression level is regulated by methyltransferase [4, 5]. Researches have shown that m7G plays a critical role in almost every stage of the life cycle of mRNA, including regulating mRNA splicing, nuclear export of mRNA, mRNA stability, translation, and transcription [6–11]. Due to the importance and particularity of the N7-methylguanosine, accurate determination of the distribution of m7G in transcriptome is the basis for the in-depth understanding of its biological functions and modification mechanisms.

Recently, studies have shown that high-throughput sequencing methods (e.g., AlkAniline-Seq [12], MeRIP-seq [13], and miCLIP-seq [14]) can be utilized to identify m7G sites. However, these methods are expensive and time-consuming for performing transcriptome-wide detections. Identifying m7G sites based on computational methods could overcome these limitations, and an accurate, effective, and efficient machine learning algorithm can predict m7G in the transcriptome. At present, researchers have proposed a few computational tools to identify m7G sites. Chen et al. [2] constructed a m7G site prediction framework called iRNA-m7G, which employed support vector machine as the classifier. And the feature vectors of the RNA sequences were extracted by fusing nucleotide property and frequency (NPF), pseudo nucleotide composition (PseDNC), and secondary structure component (SSC). Song et al. [15] proposed the m7GFinder method, which considered both sequence and genome-derived features. And support vector machine was used for prediction. Dai et al. [4] developed a m7G site predictor m7G-IFL using physical-chemical properties (PCP), binary and k-mer frequency (BKF), and ring-function-hydrogen (RFH) properties to extract features. The 10-fold cross-validation proved that m7G-IFL has better performance than iRNA-m7G, and the accuracy of the m7G-IFL reached 92.5%. Although researchers have proposed a series of calculation methods contributed to the studies of m7G site prediction, most methods only use a single traditional classifier. Moreover, accumulating evidences show that natural language processing (NLP) technique can convert biological sequences such as DNA sequences and protein sequences into feature descriptors successfully [16–21]. It is worth noting that most of the work at this stage uses static word embedding technology to convert biological sequences into feature matrices, while static word embedding technology cannot capture additional knowledge except semantic and syntactic information from the context of a sentence or paragraph.

Enlightened by this, the BERT-m7G method based on stacking ensemble classifier and bidirectional encoder representations from transformers (BERT) algorithm is proposed for m7G site prediction. Above all, BERT is used for the first time to convert RNA sequences into feature descriptors. Secondly, we construct a feature selection scheme based on the elastic net method to eliminate the redundancy and noise information in the initial feature space obtained by BERT. Finally, the optimal feature subset is input into stacking ensemble classifier whose hyperparameters are tuned with tree-structured Parzen estimator (TPE) approach which is a Bayesian optimization algorithm under the SMBO framework. In addition, the 10-fold cross-validation on benchmark dataset indicates that the method BERT-m7G proposed in this paper has better prediction performance compared with other state-of-the-art prediction methods.

## 2. Materials and Methods

### 2.1. Dataset

In this study, the benchmark dataset is downloaded from the work of Dai et al. [4] to compare the proposed computational method with other state-of-the-art prediction methods. The construction process of the dataset satisfies the following criteria: (1) The positive samples in the benchmark dataset are the sequences centered on the N7-methylguanosine site, which are detected by Drummond et al. [8] and derived from human HeLa and HepG2 cells. (2) Both positive and negative sample sequences have the same number of 741, which eliminates the impact of unbalanced dataset on the construction of robust model. And all the sample sequences are formed by 41 nucleotides with guanine in the center. (3) The CD-HIT software is employed to reduce sequence homology bias and remove sequences with more than 80% sequence similarity.

### 2.2. Feature Extraction

As an entirely bidirectional unsupervised language representation model, the outstanding performance of bidirectional encoder representation from transformers (BERT) has demonstrated in eleven NLP tasks. As it is mentioned in the original BERT paper [22], all variants of BERT are obtained after BookCorpus containing 800 million words and English Wikipedia containing 2500 million words are acted as the pretrained corpus with spending a lot of time to train. In addition, transfer learning has been extensively used in machine learning and deep learning applications. Through transfer learning, the knowledge learned from one domain or task can be transferred to fulfil tasks with less abundant data or in other domains. Thus, we attempt to use the pretrained BERT model to extract RNA sequence information because it has the following two advantages: the large amount of information is used to train BERT and the domain transfer from natural language to RNA language.

BERT, which differs from other language models, is a deep learning context representation model that can obtain deep bidirectional contextual information in RNA language. This means that using the BERT model enables the same word or nucleotide in different positions of the sentence to adopt different continuous real-valued vectors. Traditional embedding methods can only generate static distribution representations for words and cannot provide effective modeling for polysemous words nor can they provide coverage for out-of-vocabulary words. Compared with them, BERT based on the training language model has the advantages of context-dependent embedding, taking the word position into consideration and supporting for out-of-vocabulary words. Therefore, we suppose that adopting the BERT can better capture hidden information aiding to m7G site prediction.

Two new unsupervised prediction tasks are used to pretrain BERT, namely, the masked language modeling (MLM) task for capturing word-level representation and the next sentence prediction (NSP) task for capturing sentence-level representation. In order to train the deep bidirectional representations, the researchers mask 15% of the words in each sequence randomly and use information including location information to infer them. This process is called “masked language modeling” in the paper. The Google research team has released various pretrained BERT models with specific configurations. According to previous researches, it can be found that in bioinformatics tasks, using BERT-based multilingual cased pretrained model can achieve better prediction performance than using other pretrained models [23, 24]. Thus, the BERT-based multilingual cased pretrained model is chosen to perform the experiments in this study. First of all, we insert spaces between the bases of the RNA sequence to form a series of nucleotides, each of which are regarded as a word of human language, and then input them into the BERT model. Each layer in the BERT pretrained model is an encoder, and the output of the previous layer is the input of the next one. Finally, we sum up the feature vectors of the last four layers generated by BERT as the feature representation vector for each nucleotide of the studied RNA sequence. The BERT-based multilingual cased pretrained model converts each nucleotide of the sequence into a contextualized word embedding vector with a size of 768 (default size). Hence, an RNA sequence with a length of 41 nt will be converted into a 31488 (41 × 768) dimensional feature vector.

### 2.3. Feature Selection Method

Elastic net proposed by Hui and Hastie, Wang et al., and Shen et al. is a linear regression model trained with L1 and L2 norms as a prior regular term [25–27]. The mixing percentage between the L1 norm and L2 norm is controlled by the parameter *β*. The elastic net method can convert the high-dimensional data into low-dimensional data while preserving the effective data. The objective function of the elastic net can be defined as follows:

(1) $$\begin{array}{c} \underset{w}{\min}\frac{1}{2\times n}{\left\|y-Xw\right\|}_{2}^{2}-\alpha \times \beta {\left\|w\right\|}_{1}+\frac{1}{2}\alpha \times \left(1-\beta \right){\left\|w\right\|}_{2}^{2}, \end{array}$$

where *X* represents the sample matrix, *y* is the category label, *n* indicates the number of samples, *α* and *β* are the nonnegative penalty parameters, and *w* denotes the regression coefficient.

### 2.4. Stacking Ensemble Classifier

The stacking ensemble classifier is an ensemble method that uses the prediction results of multiple classifiers as new features for retraining. By integrating information from multiple prediction models, the stacking ensemble classifier can achieve the purpose of minimizing the generalization error and obtain better prediction performance than the single classifier [28–30]. In this study, we build BERT-m7G based on the stacking ensemble classifier to identify m7G sites. It mainly conducts two stages of learning. In the first stage, the matrices generated by BERT and category labels are provided to the base classifiers together. In the second stage, the metaclassifier applies the probability output values produced by the base classifiers as input for fitting and outputs the final prediction results.

For BERT-m7G, we explore six different classifiers in the first place to choose the base classifiers of the stacking ensemble classifier algorithm, including light gradient boosting machine (LightGBM) [31], support vector machine (SVM) [32], random forest (RF) [33], naive Bayes (NB) classifier [34, 35], logistic regression (LR) [36, 37], and gradient boosting decision tree (GBDT) [38, 39]. Subsequently, LightGBM, SVM, and LR are selected as optimum combination of the base classifier because when the optimal feature subset is used as the input features of classifiers, their prediction accuracy values are higher than those of other machine learning classifiers. And compared to combining other classifiers as the base classifiers, choosing LR, SVM, and LightGBM as base classifiers can obtain a better prediction accuracy value. Then, the probability output values of the first stage are used as new features, which are input to metaclassifier. BERT-m7G can mine the essential abstract features characterized m7G sites through hierarchical learning, and its prediction performance is superior to using the single classifiers.

### 2.5. Performance Evolution

In this study, 10-fold cross-validation [40] is used to assess the effectiveness of the proposed predictor. Four common measurement metrics are used to measure the prediction results, including sensitivity (SN), specificity (SP), accuracy (ACC), and Matthew's correlation coefficient (MCC) [41–43], which are defined as follows:

(2) $$\begin{array}{c} \text{SN}=\frac{\text{TP}}{\text{TP}+\text{FN}},\;\left(0\leq \text{SN}\leq 1\right), \end{array}$$

(3) $$\begin{array}{c} \text{SP}=\frac{\text{TN}}{\text{TN}+\text{FP}},\;\left(0\leq \text{SP}\leq 1\right), \end{array}$$

(4) $$\begin{array}{c} \text{ACC}=\frac{\text{TP}+\text{TN}}{\text{TP}+\text{TN}+\text{FP}+\text{FN}},\;\left(0\leq \text{ACC}\leq 1\right), \end{array}$$

(5) $$\begin{array}{c} \text{MCC}=\frac{\text{TP}\times \text{TN}-\text{FP}\times \text{FN}}{\sqrt{\left(\text{TP}+\text{FN}\right)\times \left(\text{TN}+\text{FN}\right)\times \left(\text{TP}+\text{FP}\right)+\left(\text{TN}+\text{FP}\right)}},\;\left(-1\leq \text{MCC}\leq 1\right), \end{array}$$

where TP stands for true positive, which is the number of positive samples that are predicted to be positive samples; TF indicates true negative, which is the number of negative samples that are predicted to be negative samples; FP represents false positive, which is the number of negative samples that are incorrectly predicted to be positive samples; FN indicates false negative, which is the number of positive samples that are incorrectly predicted to be negative samples. Moreover, the generalization performance of the prediction model can be reflected by the area under the receiver operator characteristic (ROC) curve [44, 45]. AUC indicates the area under the ROC curve, the closer the value of AUC is to 1, the better robustness of the model.

### 2.6. Description of the BERT-m7G Process

**Figure 1:**
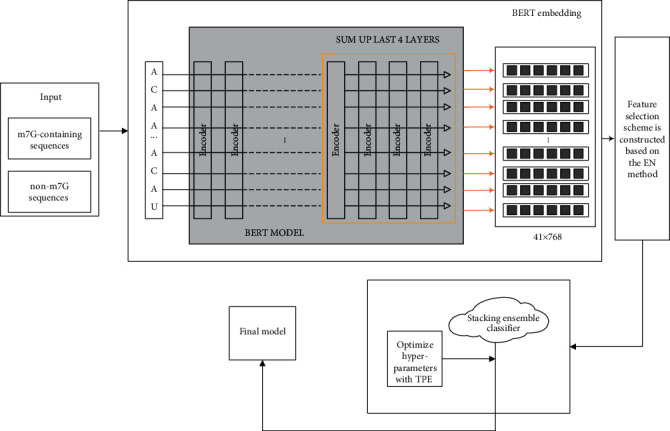
The flow chart of the BERT-m7G method.

In this study, we propose a novel method BERT-m7G, which is used to identify m7G sites. The workflow of BERT-m7G is shown in Figure 1. All experiments are performed on the Windows operating system with 32.0 GB RAM and implemented by Python 3.7 programming.

The specific steps for BERT-m7G to identify m7G sites are described as follows:

- Step 1: Data preparation. Obtain the N7-methylguanosine modification dataset, including the RNA positive sample sequences (i.e., m7G-containing sequences) and negative sample sequences (i.e., non-m7G sequences) and their corresponding class labels
- Step 2: Feature coding. First, insert a space between each base of the RNA segment and then input it into the BERT model. Finally, we sum up the feature vectors of the last four layers to construct the initial feature vector
- Step 3: Feature selection. The feature selection scheme is constructed based on the EN method, which is used to remove redundant and irrelevant information while retaining features related to classification. Firstly, we assign scores to each feature according to their coefficients in elastic net with regularization. Secondly, arrange the features in descending order according to the importance scores. Finally, the features that have zero feature coefficients are deleted
- Step 4: Model construction. In the first stage, LightGBM, SVM, and LR are selected as the base classifiers by comparing the prediction accuracy of multiple classifiers and the base classifiers combined by different classifiers on the optimal feature subset. In the second stage, the probability output values of the base classifiers are input to the metaclassifier LR and then output the final classification probabilities
- Step 5: Model optimization. In order to further improve the prediction performance of the BERT-m7G, we use the TPE algorithm to optimize the hyperparameters of the different classifiers (i.e., base classifiers and metaclassifier) constituted the stacking ensemble classifier
- Step 6: Model evaluation. We compute ACC, SN, SP, and MCC values via 10-fold cross-validation to assess the prediction performance of the model, and draw the ROC curve to evaluate the robustness of the model

## 3. Results and Discussion

### 3.1. Comparison of Different Feature Selection Methods

The features generated by BERT are used as the initial feature space for building the classification model. The redundancy and noise information in the initial feature space affect the prediction accuracy of the model and reduce the speed of calculation. Therefore, we select locally linear embedding (LLE) [46], spectral embedding (SE) [47], XGBoost [48], light gradient boosting machine (LightGBM) [49], principle component analysis (PCA) [50], Boruta [51], singular value decomposition (SVD) [50], and elastic net (EN) methods to reduce the dimensionality of the initial feature space and the difficulty of the learning task. When using EN as the feature selection method, we first assign scores to each feature based on their own coefficients in the EN with regularization, then sort the features in descending order according to the importance scores, and finally remove the features with an importance score of zero. Similarly, when performing feature selection based on the XGBoost algorithm or LightGBM algorithm, we first employ the algorithm to prioritize the features and then discard the features whose importance score is equal to zero. The Boruta method can select features aiding to classification from the feature space as the optimal feature subset. Moreover, to compare with the EN method preferably, the feature subsets corresponding to the feature selection methods LLE, SE, SVD, and PCA are set to the same feature dimensions as the EN method. The feature subsets selected by different feature selection methods are fed into the stacking ensemble classifier, whose base classifiers are LightGBM, LR, and SVM, and the metaclassifier is LR. The prediction results and the dimensions of the eight feature selection methods are shown in Table 1, in which “All” denotes the initial feature vector set without doing feature reduction and “Optimal” represents the dimension of the optimal feature subsets.

**Figure 2:**
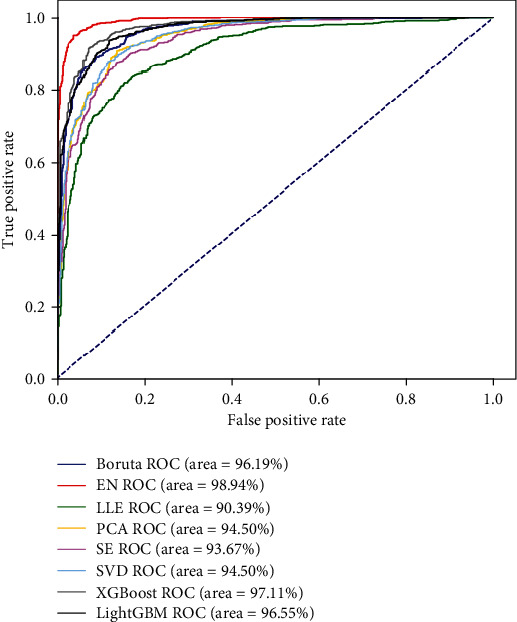
The ROC curves of the eight feature selection methods which are XGBoost, LightGBM, Boruta, SVD, PCA, SE, LLE, and EN.

It can be observed from Table 1 that the choice of different feature selection methods has a great impact on the performance of m7G site prediction model. EN has a better dimensional reduction effect compared with the other seven feature selection methods. The prediction accuracy of EN obtains the maximum value of 95.34%, which is 3.5%, 5.06%, 5.66%, 7.56%, 7.82%, 8.83%, and 12.41% higher than those of XGBoost, LightGBM, Boruta, SVD, PCA, SE, and LLE, respectively. Meanwhile, the MCC of EN obtains the maximum value of 0.9074, which is 0.0699, 0.1005, 0.1121, 0.1504, 0.1552, 0.1762, and 0.2471 higher than those of XGBoost, LightGBM, Boruta, SVD, PCA, SE, and LLE, respectively. The ACC and MCC values of the models trained on the optimal feature subsets obtained by the EN, Boruta, XGBoost, and LightGBM methods have improved compared with the model trained on the initial feature space. However, the ACC and MCC values of the models trained on the feature subsets obtained by the SVD, PCA, SE, and LLE methods have decreased compared with the situation without dimensionality reduction. In addition, we further verify the robustness and generalization ability of the prediction models with different feature selection methods through the ROC curves, which are shown in Figure 2.

It can be seen from Figure 2 that the area under the ROC curve obtained by the EN is 98.94%, which is 1.83%, 2.39%, 2.75%, 4.44%, 4.44%, 5.27%, and 8.55% higher than those of XGBoost, LightGBM, Boruta, SVD, PCA, SE, and LLE, respectively, indicating that the robustness of the m7G site prediction model constructed by the EN method is better than that constructed using other feature selection methods. Thus, considering the calculation speed and prediction performance of the model, we use the feature selection scheme which is constructed based on the EN method to remove redundant and noisy information, while retaining features that help model classification.

### 3.2. Comparison of Different Feature Extraction Methods

In order to clearly reflect the superiority of our newly proposed feature extraction method, we also choose five well-known feature extraction methods to convert RNA sequence information into numerical matrices, including trinucleotide composition (TNC) [52, 53], K-spaced nucleotide pair frequencies (KSNPFs) [54, 55], dinucleotide composition (DNC) [56, 57], nucleotide chemical property (NCP) [58–62], and accumulated nucleotide frequency (ANF) [63]; then, the feature matrices extracted by different methods are sequentially input into the model for prediction of m7G sites. The experimental results based on the 10-fold cross-validation are shown in Table 2.

**Figure 3:**
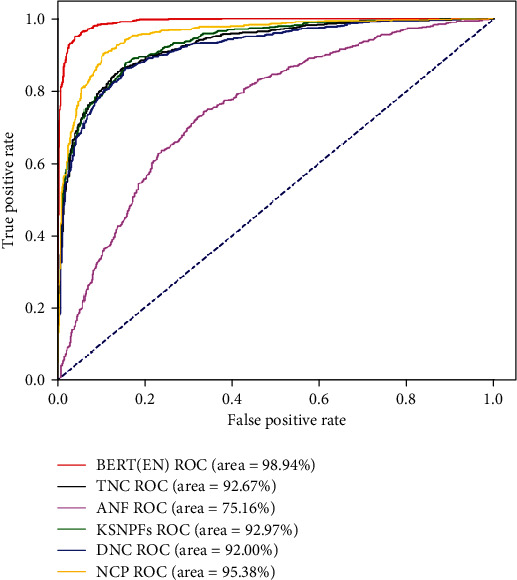
ROC curves of different feature extraction methods.

It is known from Table 2, for the five feature extraction methods including TNC, KSNPFs, NCP, DNC, and ANF, the NCP method achieves the optimal prediction performance, whose SN, SP, ACC, and MCC values reach 89.88%, 88.13%, 89.00%, and 0.7814. The ACC value of NCP is 3.44%, 4.05%, 4.25%, and 18.9% higher than those of TNC, KSNPFs, DNC, and ANF, respectively. NCP's MCC value is 0.0687, 0.0811, 0.0841, and 0.3776 higher than those of TNC, KSNPFs, DNC, and ANF, respectively. Furthermore, the model constructed by BERT (EN) has the best predictive performance, whose ACC reaches 95.34%, which is 9.78%, 10.39%, 6.34%, 10.59%, and 25.24% higher than those of TNC, KSNPFs, NCP, DNC, and ANF, respectively. BERT (EN)'s MCC value is 0.1947, 0.2071, 0.126, 0.2101, and 0.5036 higher than those of TNC, KSNPFs, NCP, DNC, and ANF, respectively. BERT (EN) represents the feature vectors generated using the feature selection scheme which is constructed based on the EN method to reduce the dimension of the initial feature space obtained by BERT. In order to assess the robustness and generalization performance of the m7G site prediction models with different feature extraction methods, we also draw the ROC curves, as illustrated in Figure 3.

From Figure 3, we can intuitively see that for the benchmark dataset, the area under the ROC curve of BERT (EN) reaches the maximum value of 98.94%, which is 6.27%, 5.97%, 3.56%, 6.94%, and 23.78% higher than those of TNC, KSNPFs, NCP, DNC, and ANF separately, revealing that the prediction model built by this method has the optimal robustness and generalization ability. The above results fully prove that using our newly proposed feature extraction method can help the model produce a better result in terms of all measurement metrics.

### 3.3. Selection of Classification Algorithms

The choice of classifier plays a key role in constructing an accurate and effective m7G site prediction model. In this study, we use BERT to convert RNA sequences into feature descriptors. Then choose the feature selection scheme which is constructed based on the EN method to sort the features and eliminate the features that have an importance score of zero, so as to obtain the optimal feature subset. Finally, the optimal feature subset is used as the input features of the model. For the sake of evaluating the effectiveness of the model proposed in this paper, we compare stacking ensemble classifier with logistic regression (LR), naïve Bayes (NB), random forest (RF), gradient boosting decision tree (GBDT), support vector machine (SVM), and light gradient boosting machine (LightGBM).  Table 3 demonstrates the prediction results for seven classifiers obtained by 10-fold cross-validation. As can be seen from Table 3, the accuracy of stacking ensemble classifier is the highest, reaching 95.34%, which is 6.07%, 0.81%, 0.87%, 6.14%, 6.95%, and 7.28% higher than those of LightGBM, SVM, LR, GBDT, NB, and RF, respectively. Meanwhile, the MCC of stacking ensemble classifier reaches the maximum value of 0.9074, which is 0.1211, 0.0162, 0.0172, 0.1226, 0.1386, and 0.1451 higher than those of LightGBM, SVM, LR, GBDT, NB, and RF, respectively. Furthermore, the SN and SP values of stacking ensemble classifier are 95.01% and 95.34%, respectively. The results of the experiment denote that stacking ensemble classifier whose base classifiers are LightGBM, LR, and SVM and the metaclassifier is LR is more suitable for identifying the sites of m7G. In addition, we try to use different classifiers as the base classifiers of stacking ensemble classifier to build the prediction model with the best performance. The comparison of the prediction results is shown in Supplementary Table S1, in which “SVM-LR-LightGBM” is used as an example; it represents the prediction model that chooses LightGBM, LR, and SVM as base classifiers and LR as the metaclassifier. From Supplementary Table S1, we can clearly see that the stacking ensemble classifier which selects LightGBM, LR, and SVM as base classifiers and LR as the metaclassifier achieves the best prediction performance.

To further improve the performance of the model, the tree-structured Parzen estimator (TPE) approach is used to optimize some critical hyperparameters of the classification model. TPE approach and Gaussian process (GP) approach are two different modeling strategies of SMBO algorithm. The Gaussian process-based approach directly models *p*(*y* | *x*), while TPE is based on *p*(*x* | *y*) and *p*(*y*) to model *p*(*y* | *x*) indirectly [64]. Moreover, we choose the expected improvement (EI) as the acquisition function to determine the local optimal hyperparameter settings. The accuracy value between the experimental value and the 10-fold cross-validation prediction value is defined as the fitness function evaluation of the hyperparameter optimization of the classification model. The optimization ranges and results of the hyperparameters are shown in Table 4. After adjusting the hyperparameters, the SN, SP, ACC, and MCC of the model are 95.82%, 95.14%, 95.48%, and 0.9100, respectively. Compared with the model without hyperparameter optimization, the prediction performance of the new model has been improved. Among them, ACC and MCC are 0.14% and 0.26% higher than the values corresponding to the model without carrying out hyperparameter optimization, respectively. Therefore, the final prediction model is constructed after hyperparameter optimization by TPE approach.

### 3.4. Comparison of BERT-m7G with Other State-of-the-Art Methods

For the sake of proving the effectiveness of the proposed BERT-m7G, we compare our method with other state-of-the-art methods for predicting m7G sites, including m7G-IFL, m7GFinder, and iRNA-m7G. Among all, iRNA-m7G proposes four models, and we compare BERT-m7G with all the models presented in iRNA-m7G. To ensure the fairness of comparison, the above prediction methods all compute the same evaluation metrics (i.e., ACC, SN, SP, and MCC) via 10-fold cross-validation on the same dataset.  Table 5 details the comparison results of BERT-m7G with existing prediction methods.

From Table 5, we can obtain that the BERT-m7G achieves the best prediction performance with an SN of 95.8%, SP of 95.1%, ACC of 95.5%, and MCC of 0.910. Compared to m7G-IFL, our model is higher by 3.4%, 2.5%, 3%, and 0.06 for SN, SP, ACC, and MCC, respectively. Moreover, the ACC value of BERT-m7G is enhanced by 5.6%, 11.1%, 5.6%, 20.7%, and 5.6% compared with the prediction methods iRNA-m7G (fusion), iRNA-m7G (PseDNC), iRNA-m7G (NPF), iRNA-m7G (SSC), and m7GFinder, respectively. Meanwhile, the MCC value is 0.112, 0.219, 0.112, 0.415, and 0.111 higher than the prediction methods iRNA-m7G (fusion), iRNA-m7G (PseDNC), iRNA-m7G (NPF), iRNA-m7G (SSC), and m7GFinder, respectively. These experimental results demonstrate that the proposed BERT-m7G method achieves an excellent prediction performance and outperforms other state-of-the-art prediction methods.

## 4. Conclusions

Since m7G plays an essential role in the regulation of gene expression, the accurate identification of m7G sites in the transcriptome is helpful to further understand their biological functions and mechanisms. In this research, we propose a new method, namely, BERT-m7G, which uses pretrained BERT model to capture hidden information aiding to m7G site prediction. For the benchmark dataset, we first adopt BERT to convert RNA sequence information into feature matrices. Then, we build the feature selection scheme based on the elastic net method to determine nonredundant and important feature subset. Finally, multiple hyperparameters of the stacking ensemble classifier are adjusted by TPE approach to obtain the best model. The 10-fold cross-validation shows that the SN, SP, ACC, and MCC of the proposed BERT-m7G are 95.8%, 95.1%, 95.5%, and 0.910, respectively. Compared with state-of-the-art methods, the ACC is advanced by 3%-20.7%, and the MCC is improved by 0.06-0.415. These experimental results indicate that our method has excellent performance for m7G site prediction.

## Figures and Tables

**Table 1:** The prediction results of different feature selection methods.

Methods	SN (%)	SP (%)	ACC (%)	MCC	Optimal
EN	**95.68**	**95.01**	**95.34**	**0.9074**	**473**
XGBoost	91.50	92.18	91.84	0.8375	753
LightGBM	90.15	90.42	90.28	0.8069	2206
Boruta	89.20	90.15	89.68	0.7953	1090
SVD	87.72	87.85	87.78	0.7570	473
PCA	87.45	87.59	87.52	0.7522	473
SE	86.24	86.78	86.51	0.7312	473
LLE	83.13	82.73	82.93	0.6603	473
All	88.66	87.73	88.19	0.7651	31488

**Table 2:** Comparison of different feature extraction methods.

Methods	SN (%)	SP (%)	ACC (%)	MCC
TNC	87.32	83.81	85.56	0.7127
KSNPFs	86.37	83.54	84.95	0.7003
NCP	89.88	88.13	89.00	0.7814
DNC	87.18	82.32	84.75	0.6973
ANF	67.20	73.00	70.10	0.4038
BERT (EN)	**95.68**	**95.01**	**95.34**	**0.9074**

**Table 3:** Performance comparison of different classifiers.

Classifier	SN (%)	SP (%)	ACC (%)	MCC
LightGBM	89.07	89.47	89.27	0.7863
SVM	93.79	95.28	94.53	0.8912
LR	94.47	94.46	94.47	0.8902
GBDT	89.34	89.07	89.20	0.7848
NB	88.80	87.99	88.39	0.7688
RF	88.94	87.18	88.06	0.7623
Stacking	**95.68**	**95.01**	**95.34**	**0.9074**

**Table 4:** Hyperparameter optimization results of stacking ensemble classifier.

	Classifier	Hyperparameters	Meaning	Search ranges	Optimal values
Base classifiers	LR	C1	The reciprocal of the regularization coefficient *λ*	(1, 50)	0.0181
	LightGBM	learning_rate	Learning rate	(0.01, 1.0)	0.2533
		max_depth	Maximum depth of the tree	(1, 50)	12
		max_bin	The max number of bins that feature values will be bucketed in	(10, 100)	84
		boosting_type	Training method	gbdt; goss; dart	gbdt
		num_leaves	Number of leaf nodes	(1, 50)	10
		n_estimators	Number of iterations	(100, 600)	255
	SVM	C2	Regularized constant which determines regularized penalty to estimation errors	(1, 50)	1.1322
		Kernel	Kernel function which uses to realize the nonlinear map from the raw feature space to high-dimensional feature space	Linear; sigmoid; poly; rbf	rbf
Metaclassifier	LR	C3	The reciprocal of the regularization coefficient *λ*	(1, 50)	35.5133

**Table 5:** The result comparison of different prediction methods.

Methods	SN (%)	SP (%)	ACC (%)	MCC
iRNA-m7G (fusion)^∗^	89.1	90.7	89.9	0.798
iRNA-m7G (PseDNC)^∗^	80.3	88.5	84.4	0.691
iRNA-m7G (NPF)^∗^	89.1	90.7	89.9	0.798
iRNA-m7G (SSC)^∗^	73.8	75.7	74.8	0.495
m7GFinder^∗^	90.8	89.1	89.9	0.799
m7G-IFL^∗^	92.4	92.6	92.5	0.850
BERT-m7G	**95.8**	**95.1**	**95.5**	**0.910**

^∗^Results excerpt from [4].

## Data Availability

The data used to support the findings of this study comes from public database, which have been cited.

## Supplementary Materials

Supplementary Materials Table S1: comparison of prediction results of different base classifier combinations of stacking ensemble classifiers.
