# Supplementary material for: BERT-m7G: A Transformer Architecture Based on BERT and Stacking Ensemble to Identify RNA N7-Methylguanosine Sites from Sequence Information
Source: Comput Math Methods Med. 2021 Aug 25;2021:7764764. doi: 10.1155/2021/7764764 (PMC8413034; doi:10.1155/2021/7764764)
Supplement: Supplementary Materials — Table S1: comparison of prediction results of different base classifier combinations of stacking ensemble classifiers. [file 7764764.f1.docx]

**Supplementary materials:**

BERT-m7G: A transformer architecture based on BERT and stacking ensemble to identify RNA N7-methylguanosine sites from sequence information

Lu Zhang^1^, Xinyi Qin^1^, Min Liu^1^, and Guangzhong Liu^1,*^ , Yuxiao Ren^2^

^1^ College of Information Engineering, Shanghai Maritime University, 1550 Haigang Ave., Shanghai 201306, China

^2^ School of computer science and engineering, Southeast University, Nanjing 214135, China

***** Correspondence: [gzhliu@shmtu.edu.cn](mailto:gzhliu@shmtu.edu.cn)

**Table of contents**

**Supplementary Tables**

**Table S1.** Comparison of prediction results of different base-classifier combinations of stacking ensemble classifiers.

**1. Supplementary Tables**

**Table S1.**

Comparison of prediction results of different base-classifier combinations of stacking ensemble classifiers.

| **Classifier** | **SN (%)** | **SP (%)** | **ACC (%)** | **MCC** |
| --- | --- | --- | --- | --- |
| SVM-LR-LightGBM | **95.68** | **95.01** | **95.34** | **0.9074** |
| SVM-LR-RF | 94.87 | 95.01 | 94.94 | 0.8994 |
| SVM-LR-GBDT | 95.55 | 94.87 | 95.21 | 0.9047 |
| SVM-LR-NB | 95.01 | 95.01 | 95.01 | 0.9007 |
| SVM-LR-LightGBM-NB | 95.01 | 94.47 | 94.74 | 0.8954 |
| SVM-LR-LightGBM-GBDT | 95.55 | 95.01 | 95.28 | 0.9061 |
| SVM-LR-LightGBM-RF | 95.55 | 95.01 | 95.28 | 0.9061 |
| SVM-LR-LightGBM-GBDT-NB-RF | 95.01 | 94.20 | 94.60 | 0.8927 |

*Note*: In order to build the prediction model with the best performance, we try to use different classifiers as the base-classifiers of stacking ensemble classifier, and choose LR as the meta-classifier to integrate the probability output values of base-classifiers and learn the relationship between different predictors and real class labels. Taking "SVM-LR-LightGBM-GBDT-NB-RF" as an example, it represents the prediction model whose base-classifiers are SVM, LR, LightGBM, GBDT, NB, and RF, and the meta-classifier is LR.
